# Cross-hybridization between HPV genotypes in the Linear Array Genotyping Test confirmed by Next-Generation Sequencing

**DOI:** 10.1186/s13000-019-0808-2

**Published:** 2019-04-22

**Authors:** Cristina Artaza-Irigaray, María Guadalupe Flores-Miramontes, Dominik Olszewski, Verónica Vallejo-Ruiz, Laura Patricia Limón-Toledo, Cibeles Sánchez-Roque, Rocío Mayoral-Torres, Luis Felipe Jave-Suárez, Adriana Aguilar-Lemarroy

**Affiliations:** 1 División de Inmunología, Centro de Investigación Biomédica de Occidente, Instituto Mexicano del Seguro Social (IMSS), Sierra Mojada No. 800, Col. Independencia, 44340 Guadalajara, Jalisco Mexico; 2 Institute of Pharmacy and Molecular Biotechnology, University of Heidelberg, Heidelberg, Germany; 3 Centro de Investigación Biomédica de Oriente, IMSS, Metepec, Puebla Mexico; 4 Clínica de Displasias, UMAE - Hospital de Ginecología y Obstetricia, Centro Médico Nacional de Occidente, IMSS, Guadalajara, Jalisco Mexico; 5 Hospital Civil de Guadalajara, Dr. Juan I. Menchaca, Guadalajara, Jalisco Mexico; 6 Joint Master Program in Neuroscience, Université de Strasbourg, Strasbourg, France

**Keywords:** Cervical samples, HPV, L1 mutations, Linear Array HPV Genotyping Test, Next-Generation Sequencing, Cross-hybridization

## Abstract

**Background:**

Linear Array Genotyping Test (LA) is one of the gold standards used for Human Papillomavirus (HPV) genotyping, however, since its launching in 2006, new HPV genotypes are still being characterized with the use of high specificity techniques such as Next-Generation Sequencing (NGS). Derived from a previous study of the IMSS Research Network on HPV, which suggested that there might be cross-reaction of some HPV genotypes in the LA test, the aim of this study was to elucidate this point.

**Methods:**

Double stranded L1 fragments (gBlocks) from different HPVs were used to perform LA test, additionally, 14 HPV83+ and 26 HPV84+ cervical samples determined with LA, were individually genotyped by NGS.

**Results:**

From the LA HPV83+ samples, 64.3% were truly HPV83+, while 42.9% were found to be HPV102+. On the other hand, 69.2% of the LA HPV84+ samples were HPV84+, while 3.8, 11.5 and 30.8% of the samples were indeed HPV 86, 87 and 114 positive, respectively. Additionally, novel nucleotide changes in L1 gene from HPV genotypes 83, 84, 87, 102 and 114 were determined in Mexican cervical samples, some of them lead to changes in the protein sequence.

**Conclusions:**

We demonstrated that there is cross-hybridization between alpha3-HPV genotypes 86, 87 and 114 with HPV84 probe in LA strips and between HPV102 with HPV83 probe; this may be causing over or under estimation in the prevalence of these genotypes. In the upcoming years, a switch to more specific and sensitive genotyping methods that detect a broader spectrum of HPV genotypes needs to be implemented.

## Background

Human Papillomaviruses (HPV) belong to a diverse group of small non-encapsulated dsDNA viruses that have evolved over millions of years [1]. HPV classification and nomenclature is defined by the International Committee for the Taxonomy of Viruses (ICTV) and the HPVs described to date are classified in different genera and species based on L1 gene sequence percentages of identity [2]. HPVs belong to five different genera called Alpha-, Beta-, Gamma-, Mu- and Nu-PVs [3]. Each HPV is adapted to infect a specific epithelial tissue such as skin or mucosa, and most of them do not cause an apparent pathology and are cleared in some months or years. However, some Alpha HPV genotypes are medically relevant because of their carcinogenic potential if the infection persists over the years. According to the International Agency for Research on Cancer (IARC), 12 HPV genotypes are defined as oncogenic or high-risk (HR-HPVs): 16, 18, 31, 33, 35, 39, 45, 51, 52, 56, 58 and 59. Some other genotypes are possibly carcinogenic while most of the remaining viruses are non-carcinogenic or low-risk (LR-HPV) [2, 4]. HPV16, the most prevalent genotype worldwide, is found in around 60% of cervical cancer samples according to a meta-analysis performed in 115,789 HPV-positive women, followed by HPV18 and HPV45 [5].

Cervical cancer (CC) is the most common HPV-related cancer and consequently HR-HPVs are widely studied to better understand and treat HPV-driven cervical carcinogenesis. However, there are not so many studies on LR-HPVs and HPV coinfections, which could unravel important information on their role in either carcinogenesis or viral clearance. In industrialized countries, CC screening is performed with three main methods: cytology, DNA or RNA detection of HR-HPVs or cytology-HPV co-testing [4]. HPV genotyping tests are widely used worldwide in many laboratories and there is an increased need to develop reliable and cheaper tests that detect most of the high and low-risk HPVs described to date. The Linear Array Genotyping test from Roche (LA), launched in 2006, allows the detection of 37 HPV genotypes (6, 11, 16 18, 26, 31, 33, 35, 39, 40, 42, 45, 51, 52, 53, 54, 55, 56, 58, 59, 61, 62, 64, 66, 67, 68, 69, 70, 71, 72, 73 (MM9), 81, 82 (MM4), 83 (MM7), 84 (MM8), IS39, and CP6108) and is one of the gold standards used for genotyping [6–8]. Moreover, while new HPV genotypes are still being characterized, the use of high-throughput screening technologies such as Next-Generation Sequencing (NGS), allows genotyping of a broader spectrum of HPVs when compared to the commercially available tests. In a previous study of our research group, LA and 454 NGS were performed in cervical samples from Mexican women. In some of the samples, not all HPV genotypes detected with LA were confirmed by NGS (indicating the possible non-specific detection of the LA), whereas five HPVs that cannot be detected with the LA test were detected by NGS: 32, 44, 74, 102 and 114 [9]. This finding suggested that there could be a cross-reaction between those HPVs not included in LA and the probes of the test.

The aim of the present study was to determine how specific is the worldwide used LA test, by looking at a possible cross-hybridization in the LA strips and to estimate the frequency of the HPVs that could cross-react. In this work, mutations in the PGMY11/09 amplified L1 gene fragment from the HPV genotypes that cross-hybridized in LA test were described, to see if new variants or subtypes of those HPVs could be infecting the Mexican population.

## Methods

### Sample collection

In the present study, cervical samples were collected from women who attended a medical examination at the Dysplasia Clinic - Centro Médico Nacional de Occidente (CMNO-IMSS) in Guadalajara, Jalisco, Mexico; and from diverse Hospitals around Mexico as previously published [9, 10]. The sample recruitment was done from 2014 to 2017 by gynecologists, with a cytobrush inserted into the endocervical canal. From those samples, 535 were positive to HPV by using Linear Array Genotyping Test.

### HPV genotyping by Linear Array

Total DNA was extracted from cervical samples collected in Preserv-Cyt medium solution (Cat. no. 70097-002, Hologic, Inc., Marlborough, MA, USA) and purified using the AmpliLute Liquid Media Extraction Amplicor kit (Cat. no. 03750540190; Roche Molecular Systems, Inc., Branchburg, NJ, USA.) following the manufacturer instructions. All samples were genotyped by Linear Array HPV Genotyping Test (Cat. no. 03378179190, Roche Molecular Systems) as previously described [9].

### Linear Array test using L1 gBlocks

Double stranded genomic blocks (gBlocks) were designed by choosing the PGMY11/09 amplified L1 sequences from HPV reference genotypes 32 (X74475), 44 (U31788), 74 (AF436130), 86 (AF349909), 87 (AJ400628), 102 (DQ080083) and 114 (GQ244463), and were synthetized by IDT (Integrated DNA Technologies, Coralvillle, Iowa, USA). The gBlocks were resuspended in 1X TE, and subsequently 10 pg of each specific gBlock were taken to proceed with LA.

### Next-generation sequencing

The positive samples to HPV83 and HPV84 determined by LA that showed a clear hybridization band in the HPV strips, were selected to perform NGS. First, the DNA of each sample was amplified by conventional PCR with the PGMY primers [11]. Then, a second PCR reaction was performed using the same primers coupled to a universal tail sequence, as previously described [9]. Briefly, the PCR products were quantified with the fluorometry assay Qubit dsDNA HS (Cat. no. Q32854, Life Technologies, Eugene, OR, USA), subsequently, each sample was diluted to 5x10^9^ molecules per microliter. Then, individual barcodes were added to each sample using the 454 MIDs from Multiplicom NV CFTR kits (Cat. no. ML-0008.192, ML-0016.192 and ML-0124.192, Molecular Diagnostics, Niel, Belgium). Each sample was purified with the Agentcourt AMPure XP beads (Cat. no. A63880, Beckam Coulter Genomics, Danvers, MA, USA) and evaluated with the Agilent DNA 1000 kit (Cat. no. 5067-1504, Agilent Technologies, Santa Clara, CA, USA) on the 2100 Bionalyzer (Cat. no. G2939BA, Agilent Technologies). For sequencing, GS Junior Titanium kits were used in a GS Junior Sequencer, following the manufacturer’s instructions (Cat. no. 05996562001, 05996589001, 05996597001, 05996619001, Roche Diagnostics, Basel, Switzerland).

### Data analysis

The sequencing data were first analyzed with the FastQC tool to determine the quality of the sequences [12]. Then, to identify specific HPV genotype sequences present in each cervical sample, the Roche GS Reference Mapper v3.0 software was used, taking as references all the L1 sequences reported in the Papillomavirus Episteme (PaVE) database [13]. The parameters considered for mapping were the following: trimming of adapters and primers, 90% of minimum overlap identity, Phred score ≥ 20, minimum read length of 100 bp and exclusion of all the repeated reads. All HPV raw sequence reads were uploaded in the NCBI Sequence Read Archive (accession number SRP130362).

Among the nucleotide changes observed in the L1 gene from HPVs 83 (AF151983), 84 (AF293960), 86 (AF349909), 87 (AJ400628), 102 (DQ080083) and 114 (GQ244463), we described those that were present in all the reads from a specific HPV with a minimum depth of 20 reads or those found in two or more distinct samples (independently of the reads number).

Finally, a phylogenetic tree was constructed with L1 consensus sequences from HPV83, 84, 86, 87, 102 and 114 from each sample, including the L1 reference sequences from all alpha3-papillomaviruses. Evolutionary analyses were conducted in MEGA 7 software by using the muscle algorithm for the alignment of sequences and the evolutionary history was inferred by using the Maximum Likelihood method based on the Tamura-Nei model [14, 15]. All positions with less than 95% site coverage were eliminated and the tree with the highest log likelihood was chosen.

## Results

### Cross-hybridization between HPV genotypes in Linear Array Genotyping test

**Fig. 1:**
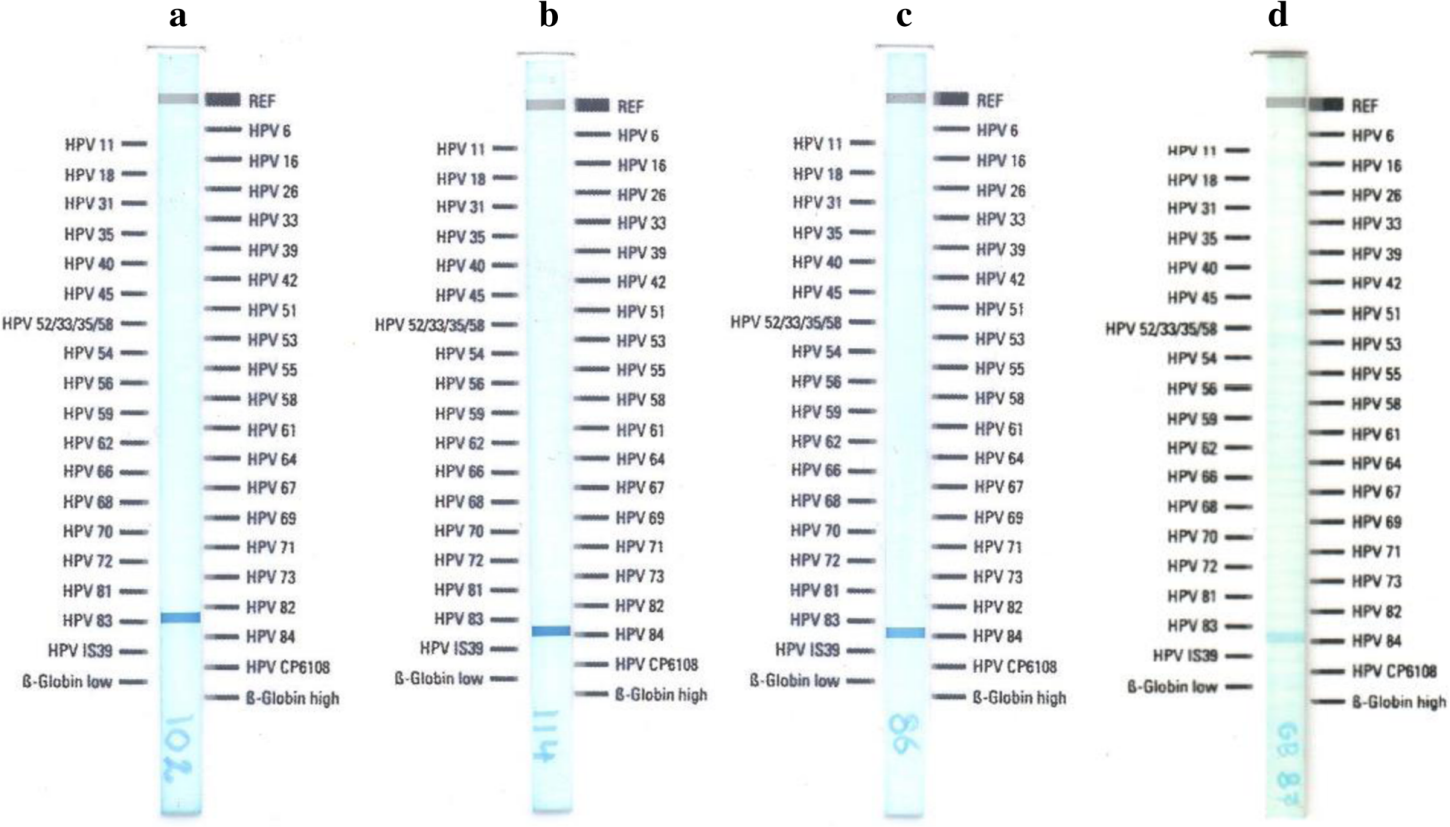
*Linear array Genotyping test* performed individually with different L1 gBlocks (double stranded genomic blocks). The gBlocks were designed from HPV genotypes 102 (**a**), 114 (**b**), 86 (**c**) and 87 (**d**) and taken as template for the LA test

Previous results of our working group showed that in some cervical samples, not all HPV genotypes detected with LA were found by NGS; in contrast, other genotypes such as 32, 44, 74, 102 and 114 were detected only with NGS. These findings suggested that positivity to some HPVs in LA could be due to the presence of other genotypes not included in the test. To see if there is a cross-reaction between HPVs 32, 44, 74, 102 and 114 (not included in the LA test) with any probe of the LA strip, amplification and hybridization was performed using as template the gBlocks of an L1 fragment from each of those HPVs. HPV32, 44 and 74 did not cross-hybridize with any of the HPV probes included in LA test (data not shown). However, L1 gBlocks from alpha-3 HPV102 and HPV114 cross-linked with HPV83 and HPV84 probes, respectively (Fig. 1a and b). Since also HPVs 86 and 87 are alpha-3 genotypes not included in LA, the test was therefore performed additionally with HPV86-L1 and HPV87-L1 gBlocks. Both HPVs showed cross-reactivity with HPV84 (Fig. 1c and d).

### Next-generation sequencing of HPV83 and HPV84 positive samples

From a total of 535 cervical samples genotyped by LA, 16 were HPV83+ (3.0%) and 35 were HPV84+ (6.5%). As HPV83 and HPV84 probes in LA strips cross-hybridize with L1 gBlocks from other genotypes, we selected 14 HPV83+ (S9, S27-S39) and 26 HPV84+ (S1-S26) samples (based on their strong hybridization band in the LA strips) to be genotyped by NGS individually. As depicted in Table 1, HPV genotyping by NGS showed that from the 26 cervical samples LA HPV84+, 17 were truly HPV84+ (65.4%), 5 were HPV114+ (19.2%), and 1 was HPV86+ (3.8%). Additionally, 2 samples were positive to both HPV 87 and 114, and finally 1 was HPV 84, 87 and 114 positive. HPV87 was found in the three cases in coinfection with HPV114. Concerning the 14 cervical samples LA HPV83+, 8 were truly HPV83+ (57.1%), 5 were HPV102+ (35.7%) and 1 was positive to both genotypes (Table 2).

**Table 1:** HPV genotyping by NGS of 26 cervical samples LA HPV84+

Sample Number	Linear Array	NGS
S1	39, 51, 56, 58, 62, **84**	39, 51, 58, 62, **84**
S2	45, 59, 66, 73, **84**, 89	45, 59, 66, 73, 89, **114**
S3	6, 53, 61, **84**, 89	6, 53, 61, **84**, 89
S4	16, 42, 45, 54, **84**	16, 69, **84**
S5	31, 56, 66, 82, **84**	56, **84**
S6	58, 81, 82, **84**, 89	16, 44, 53, 58, 66, 72, 81, 82, **84**, 89
S7	11, 42, 61, **84**	11, 61, **114**
S8	16, 51, 54, **84**	16, 54, **84**
S9	16, 53, 83, **84**	16, 53, 62, 83, **114**
S10	16, 61, **84**, 89	16, 61, 83, **84**, 89
S11	35, 66, **84**, 89	32, 35, 66, **84**, 89
S12	39, 54, 61, **84**	39, 51, 54, 58, 61, 62, **84**, **87**, 106, **114**
S13	39, 61, **84**, 89	61, 89, **87**, **114**
S14	56, 66, 70, **84**	16, 66, 70, **87**, **114**
S15	69, 71, 81, **84**	69, 71, 81, **114**
S16	11, 39, **84**	11, 39, **114**
S17	45, 62, **84**	45, 62, **84**
S18	56, 61, **84**	6, 56, 61, 68, **84**
S19	16, **84**	16, 70, 81, 83, **86**
S20	18, **84**	18, **84**
S21	31, **84**	31, 83, **84**
S22	31, **84**	31, 54, **84**
S23	45, **84**	45, **84**
S24	45, **84**	45, 66, **84**
S25	59, **84**	59, **84**
S26	66, **84**	66, **84**

The first column shows LA results and the second column NGS results. HPV84, 86, 87 and 114 are darkened for an easier visualization

**Table 2:** HPV genotyping by NGS of 14 cervical samples LA HPV83+

Sample Number	Linear Array	NGS
S27	16, 42, 58, 62, 81, **83**	16, 58, 81, **83**
S28	51, 54, 58, 66, **83**, 89	58, 66, **83**, 89
S29	11, 52, 59, 62, **83**	11, 52, 59, 62, **83**
S30	35, 54, 70, **83**, 89	35, 54, 70, 89, **102**
S31	54, 55, 61, **83**, 89	61, 89, **102**
S32	11, 62, 70, **83**	16, 61, 62, 70, **83**, 89
S9	16, 53, **83**, 84	16, 53, **83**, 114
S33	31, 62, 72, **83**	31, 72, **102**
S34	45, 59, 64, **83**	52, 56, 59, 61, 71, 74, **102**
S35	52, 61, 71, **83**	52, 61, 71, 56, 74, **102**
S36	31, 61, **83**	31, 61, **83**
S37	52, 81, **83**	52, 81, **83**
S38	51, **83**	51, 61, **83**, **102**
S39	**83**	**83**, 81

The first column shows LA results and the second column NGS results. HPV83 and 102 are darkened for an easier visualization

### L1 gene mutations from HPV83, 84, 87, 102 and 114

After comparing the sequences obtained by NGS with the L1 reference genes (downloaded from PaVE database), nucleotide variations in L1 fragment from HPV genotypes 83, 84, 87, 102 and 114 were determined. Concerning HPV83-L1, only sample S27 contained an L1 gene fragment identical to the reference, and rest of the samples showed diverse synonymous mutations. The most common nucleotide changes were c.1200A>G (in 6 samples), c.1191A>G (in 5 samples), c.1227G>C (in 5 samples), and c.1008A>G (in 4 samples) (Table 3). Regarding HPV84-L1, seventeen out of eighteen samples showed one or more nucleotide changes, being the non-synonymous mutation c.1323C>A (p.D441E) the most prevalent one (in 16 samples). Another HPV84-L1 non-synonymous mutation was detected in only one sample: c.1055A>C (p.N352T) (Table 4). Additionally, two L1 synonymous mutations were found in the three HPV87+ samples: c.1056T>C and c.1359A>G. About HPV102-L1 nucleotide changes, three out of the six positive samples (S30, S34 and S35) showed c.1362A>G mutation in their L1 sequence and only S30 sample showed the non-synonymous mutation c.1060G>A (p.G354S). Among the eight HPV114+ samples, nine mutations were detected, three of them present in all the HPV114-L1 sequences: c.1318C>G (p.P440A), c.1332A>G and c.1359C>T. Furthermore, two non-synonymous mutations were found in four samples: c.1297T>A (p.S433T) and c.1331C>A (p.T444K) (Table 5). Finally, no HPV86-L1 mutations were detected.

**Table 3:** Nucleotide changes identified in HPV83-L1 when compared to the reference sequence (AF151983) in nine HPV83+ samples

Sample number	c.1008 A > G	c.1104 A > C	c.1152 G > T	c.1170 A > C	c.1191 A > G	c.1200 A > G	c.1227 G > C	c.1227 G > A	c.1233 C > T	c.1264 C > T
S9	●				●	●	●			
S27										
S28				●						●
S29	●				●	●	●			
S32				●						●
S36		●	●			●		●	●	●
S37	●				●	●	●			
S38					●	●	●			
S39	●				●	●	●

**Table 4:** Nucleotide changes identified in HPV84-L1 when compared to the reference sequence (AF293960) in eighteen HPV84+ samples

Sample number	c.1011 A > G	c.1055 A > C^a^	c.1173 C > A	c.1323 C > A^a^
S1				●
S3				●
S4				
S5				●
S6				●
S8				●
S10				●
S11				●
S12				●
S17				●
S18	●			●
S20		●		●
S21				●
S22				●
S23			●	
S24				●
S25	●			●
S26	●			●

^a^non-synonymous nucleotide changes

**Table 5:** Nucleotide changes identified in HPV114-L1 when compared to the reference sequence (GQ244463) in eight HPV114+ samples

Sample number	c.1059 C > T	c.1167 A > C	c.1297 T > A^a^	c.1299 C > A	c.1305 G > A	c.1318 C > G^a^	c.1331 C > A^a^	c.1332 A > G	c.1359 C > T
S2			●		●	●	●	●	●
S7			●	●		●	●	●	●
S9			●	●		●	●	●	●
S12	●	●				●		●	●
S13			●		●	●	●	●	●
S14	●	●				●		●	●
S15	●	●				●		●	●
S16						●		●	●

^a^non-synonymous nucleotide changes

**Fig. 2:**
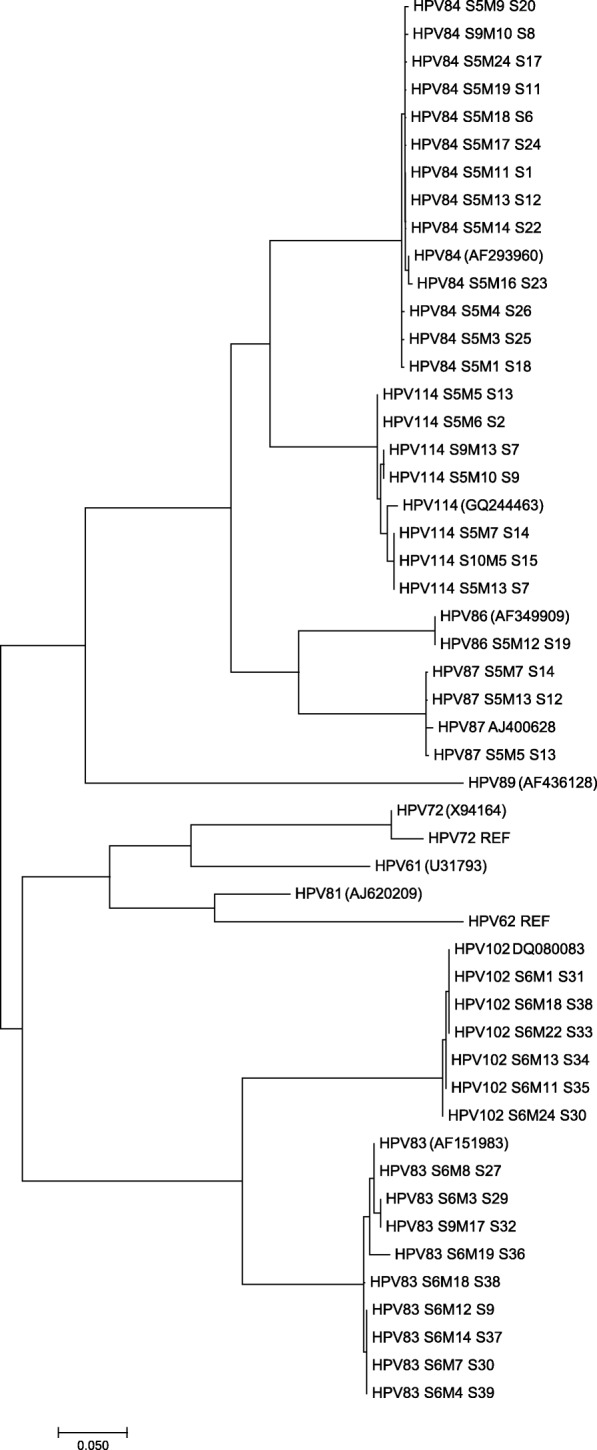
Phylogenetic tree based on alpha-3 HPVs L1 sequences. The analysis involved the 11 reference HPVs from alpha-3 species (REF) and 39 L1 sequences from HPVs 83, 84, 86, 87, 102 and 114 that infect Mexican woman. The tree is drawn to scale, with branch lengths measured in the number of substitutions per site

To visualize the relationship between the reference alpha-3 HPV genotypes and those obtained from the Mexican cervical samples (HPV83, 84, 86, 87, 102, 114), a phylogenetic tree was built based on their L1 gene sequences (Fig. 2).

## Discussion

During the last decade, cytology-based screening is being replaced with HPV genotyping; Linear Array genotyping test is commonly used in many laboratories for HPV detection [8, 16]. LA test, launched in 2006, is a PCR-based strategy with pooled primer sets, coupled to hybridization to specific probes for 37 anogenital HPV genotypes immobilized on a nylon strip [7]. This test has the most abundant data in peer-reviewed literature classifying it as one of the most frequently used [17]. Undoubtedly, LA is a sensitive test of great value for epidemiological studies and HPV genotyping, but more sensitive, specific and accurate methods such as NGS allow the identification of not only a broader spectrum of HPVs, but also subtypes and variants [18–20].

HPV84 was characterized as a novel genotype in 2001 by M. Terai and R. D. Burk (accession number AF293960) from a cervicovaginal sample obtained from a 21-year-old Caucasian female with a normal Pap smear [21]. It is considered as a LR-HPV and is among the most prevalent LR-HPVs found worldwide in cervical samples [22–30]. Both HPV83 and HPV84 belong to the Alphapapillomavirus genus, species 3 group (alpha-3) together with HPV 61, 62, 72, 81, 86, 87, 89, 102 and 114. From these genotypes, only 102 and 114 were characterized after LA test was launched [31, 32], and are obviously not included in the test, neither do HPV86 and HPV87.

In the present study, non-specific hybridization of four alpha-3 HPVs in the LA test, the golden test used to detect HPVs in a patient’s sample, is being reported. Indeed, HPV102-L1 cross-reacts with the probe that detects HPV83, and likewise, HPV genotypes 86, 87 and 114 cross-react with the probe that detects HPV84. These results explain the observations in a previous study where we reported that not all HPV genotypes detected in some cervical samples with LA test were confirmed by NGS (like HPV83 and HPV84), whereas five HPVs not included in LA were detected by NGS (HPVs 32, 44, 74, 102 and 114) [9]. With the current study, we reveal that what happened in the previous work was that positivity to HPV83 and HPV84 in the LA test was only true in the 57.1% and 65.4% of the cases, respectively. In addition, as LA test is sometimes showing positivity to HPVs 83 and 84 when they are not present in the sample, the prevalence of these genotypes could be overrated, as discussed later. Moreover, we cannot dismiss the possibility that more HPV genotypes from other species could also cross-react with some of the probes in the LA strip. An already reported limitation of LA is that additional testing is necessary to detect the HR-HPV52 when HPVs 33, 35 and/or 58 are present in the sample [33]. However, to our knowledge, this is the first report proving a cross-hybridization in LA test.

In this work, HPV genotyping by NGS of HPV84 positive samples (detected by LA), revealed that 69.2% of the samples were truly HPV84+, while 30.8% were HPV114+, 3.8% were HPV86+ and 11.5% were HPV87+. It is important to mention that some samples were coinfected with more than one of these genotypes. On the other hand, among HPV83+ samples with LA, 64.3% were confirmed as HPV83+ with NGS, while 42.9% were HPV102+. A recent publication shows that the clinical performance of the LA test within the VALGENT-3 framework, which is designed for comprehensive comparison and clinical validation of HPV tests, is accurate for primary cervical cancer screening [34]. Nevertheless, another recent genotyping study that compares ion torrent-next generation sequencing vs. linear array to detect anal HPVs in individual clinical specimens shows that the NGS assay detects 10 HPV genotypes that are not among the ones detected in LA test: 30, 32, 43, 44, 74, 86, 87, 90, 91, 114 [35]. Some of these HPVs could be cross-reacting with LA test as we demonstrated in this work.

New HPV genotyping tests must be developed in the upcoming years since an increasing number of genotypes are being described by using NGS platform, and their prevalence and role in cervical lesion progression needs to be assessed. Indeed, 105 putative new papillomavirus types have been identified by using a novel protocol based on improved PCR protocols combined with NGS [36]. Moreover, HPV coinfections could be hiding essential information on cervical lesion progression and more meticulous studies on multiple infections are also necessary in the near future for understanding the link between coinfections and carcinogenesis. Furthermore, FAP primers based sequencing is sensitive and effective for detection of cutaneous HPVs and its use in cervical samples could contribute to a broader knowledge on HPV infections in cervical mucosa [37–39].

The L1 gene is used for the classification of HPV genotypes as it is well conserved among all of them. HPV genotypes share between 71 and 89% of nucleotide identity, and within the same HPV type, HPV variants can be described [40, 41]. After HPV genotyping by NGS, new nucleotide changes in PGMY amplified L1 sequence have been described in this work for HPV genotypes 83, 84, 87, 102 and 114. From all the nucleotide changes reported here, HPV84-L1, HPV102-L1 and HPV114-L1 have two, one and three non-synonymous mutations, respectively. To our knowledge, there is only one study that also describes one of the nucleotide changes reported in this work: c.1323C>A in HPV84-L1 [42]. Further functional analysis should be done to understand the biological significance of the amino acid changes.

## Conclusions

This study showed that HPV genotypes 86, 87 and 114 cross-hybridize with HPV84 in LA test and that HPV102 cross-reacts with HPV83 probe; these observations were confirmed by comparing LA genotyping with NGS data. Therefore, there is an over estimation of HPV 83 and 84 prevalence when HPV genotyping is performed with LA. On the other hand, it would be important to determine the prevalence and role in carcinogenesis of HPVs 86, 87, 102 and 114. Novel nucleotide changes in L1 gene from HPVs 83, 84, 87, 102 and 114 were determined in Mexican cervical samples, some of them lead to changes in the protein sequence. The biological significance of those changes should be determined in the near future. There is no doubt that more specific and sensitive tests to detect a broader spectrum of HPV genotypes should be implemented in the clinical practice.

## Abbreviations

CC: Cervical Cancer
gBlocks: Genomic Blocks
HPV: Human Papilloma Virus
HR-HPV: High Risk HPV
IARC: International Agency for Research on Cancer
ICTV: International Committee for the Taxonomy of Viruses
LA: Linear Array
LR-HPV: Low Risk HPV
NGS: Next-Generation Sequencing
PaVE: Papillomavirus Episteme
